# A Shotgun Proteomic Platform for a Global Mapping of Lymphoblastoid Cells to Gain Insight into Nasu-Hakola Disease

**DOI:** 10.3390/ijms22189959

**Published:** 2021-09-15

**Authors:** Antonella De Palma, Anna Maria Agresta, Simona Viglio, Rossana Rossi, Maura D’Amato, Dario Di Silvestre, Pierluigi Mauri, Paolo Iadarola

**Affiliations:** 1 Proteomics and Metabolomics Unit, Institute for Biomedical Technologies (ITB-CNR), 20054 Milan, Italy; agresta@alpex.com (A.M.A.); rossana.rossi@itb.cnr.it (R.R.); dario.disilvestre@itb.cnr.it (D.D.S.); 2 Biochemistry Unit, Department of Molecular Medicine, University of Pavia, 27100 Pavia, Italy; simona.viglio@unipv.it (S.V.); maura.damato90@gmail.com (M.D.); 3 Biochemistry Unit, Department of Biology and Biotechnologies “L. Spallanzani”, University of Pavia, 27100 Pavia, Italy; paolo.iadarola@unipv.it

**Keywords:** Nasu-Hakola Disease, frontotemporal dementia, TREM2, proteomics, MudPIT, Lymphoblastoid cells

## Abstract

Nasu-Hakola Disease (NHD) is a recessively inherited systemic leukodystrophy disorder characterized by a combination of frontotemporal presenile dementia and lytic bone lesions. NHD is known to be genetically related to a structural defect of TREM2 and DAP12, two genes that encode for different subunits of the membrane receptor signaling complex expressed by microglia and osteoclast cells. Because of its rarity, molecular or proteomic studies on this disorder are absent or scarce, only case reports based on neuropsychological and genetic tests being reported. In light of this, the aim of this paper is to provide evidence on the potential of a label-free proteomic platform based on the Multidimensional Protein Identification Technology (MudPIT), combined with in-house software and on-line bioinformatics tools, to characterize the protein expression trends and the most involved pathways in NHD. The application of this approach on the Lymphoblastoid cells from a family composed of individuals affected by NHD, healthy carriers and control subjects allowed for the identification of about 3000 distinct proteins within the three analyzed groups, among which proteins anomalous to each category were identified. Of note, several differentially expressed proteins were associated with neurodegenerative processes. Moreover, the protein networks highlighted some molecular pathways that may be involved in the onset or progression of this rare frontotemporal disorder. Therefore, this fully automated MudPIT platform which allowed, for the first time, the generation of the whole protein profile of Lymphoblastoid cells from Nasu-Hakola subjects, could be a valid approach for the investigation of similar neurodegenerative diseases.

## 1. Introduction

Based on the last report edited by Alzheimer’s Disease International [1], every 3.2 s a person falls ill worldwide with “dementia” [2], an umbrella term that indicates a broad category of brain diseases linked to cognitive decline. These include Alzheimer’s Disease (AD), Vascular Dementia (VD), Lewy Bodies Dementia (LBD) and Frontotemporal Dementia (FTD). In 5–15% of dementia cases, FTD is the second most common cause of presenile dementias. FTD includes a large spectrum of pathologies caused by atrophy of the frontal and/or anterior temporal lobes of the brain which leads to changes in behavior, social conduct, language or speech [3]. Despite a wealth of studies on FTD, much about the molecular mechanism of the disease, including the causes of the sporadic or rare forms, remains unknown. The overlap of several symptoms with other neurological syndromes makes this category even more puzzling.

In this context, a prominent position is occupied by Nasu-Hakola Disease (NHD) or Polycystic Lipomembranous Osteodysplasia with Sclerosing Leukoencephalopathy (PLOSL), a frontotemporal neuropathology characterized by a combination of dementia and cyst-like osseous lesions. The contemporary impairment of nervous and skeletal body systems results in a disorder that is truly unique in the neurodegeneration field [4]. Despite PLOSL pathogenesis still being unknown, the clinical history of patients is well-standardized. It follows four sequential stages: a latent stage which moves to an osseous stage, an early neurological stage, and a late neurological stage that leads patients to death, usually within around 45 years [5]. With roughly two hundred cases described worldwide, NHD is likely to be the least common neuropathy, the highest incidence being reported within the Finnish and Japanese populations [6]. The genetic studies on NHD suggest that this disorder is caused by a structural defect of two genes encoding different subunits of the same membrane receptor signalling complex, specifically the DNAX-activating protein 12 (*DAP12*, also named *TYROBP*) and the Triggering Receptor Expressed on Myeloid cell 2 (*TREM 2*) genes [7,8]. *TREM2/DAP12* are expressed in different tissues including the central nervous system (CNS), microglia, pre-osteoclast and the immune system (natural killer cells, lymphocytes, macrophages and dendritic cells). The expression and distribution of this complex in brain tissue cells has been described by Sessa et al. [9]. While several *DAP12-TREM2* mutations that cause NHD onset [10] are well-identified, the precise molecular mechanism and the relationship between neuro and bone involvement in its progression are not fully understood. Thus, assuming that the purpose of scientists in this field is the development of a tailored therapy to halt/slow down the progression of illness, a great deal of research is certainly needed.

To this purpose, in an effort to uncover their protein profiles, proteomics were applied for the first time by our team to lymphoblastoid cells (LCLs) from NHD subjects [11]. LCLs from the components of an Italian family (two patients with homozygous C-to-T mutation at position 97 in exon 2 of *TREM2* gene, four patients with heterozygous mutation, and one healthy individual) were analyzed by two-dimensional electrophoresis (2-DE) followed by liquid chromatography mass spectrometry (LC-MS/MS). This resulted in the identification of 21 proteins (involved in glucose metabolism and information pathways as well as in stress responses) that were differentially expressed among groups [11]. The lack of identification of other proteins involved in the same and/or in other pathways this information, while being of great interest, suggests that their roles were clearly partial. The aim of the present work was to fill this gap by identifying most of the proteins that could discriminate against the above subjects and illustrate the metabolic pathways involved in the development/progression of this anomalous frontotemporal dementia. The application of a label-free approach, consisting of a two-dimensional liquid chromatography coupled to tandem mass spectrometry (μ2DLC-MS/MS), allowed us to achieve this goal. To the best of our knowledge, this is the first LC-MS study dealing with the investigation of NHD individuals from the same family that led to the identification of protein differences among them. Taken together, these data provide a picture of physio/pathological NHD states that could be considered as a model for studying similar neurodegenerative diseases.

## 2. Results

### 2.1. Protein Profiling

The application of a gel-free proteomic platform, based on a bi-dimensional μliquid-chromatographic system combined to a high resolution mass spectrometer (also known as MudPIT) [12,13], allowed the identification of 10,253 unique peptides and 3458 distinct proteins from a total of 19 protein lists (including biological and technical replicates), by analyzing Lymphoblastoid cell lines (LCLs) obtained from the healthy (Wt), heterozygous (He) and homozygous (Ho) components of the family indicated in the Materials and Methods section. The complete list of proteins identified for each group is reported in Table S1 of the Supplementary Material.

**Figure 1:**
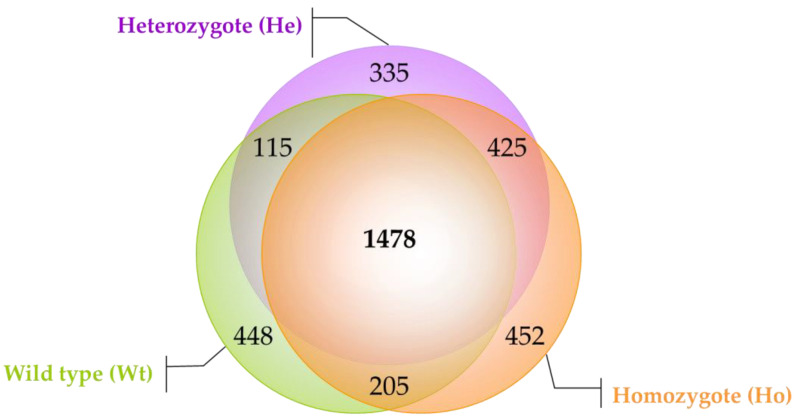
Venn Diagram. The figure shows the distribution of all identified proteins within three investigated categories (Wild type, Wt, green circle; Heterozygote, He, violet circle; Homozygote, Ho, orange circle). About 1400 proteins were shared between the three groups of subjects and approximately 400 proteins were identified as anomalous of each category.

To verify the repeatability of the data and the efficiency of our procedure, the spectral counts (SpCs), i.e., the number of mass spectra assigned to each identified protein in the first technical replicate were plotted vs the SpCs of the one identified in the second technical replicate. The same procedure was applied to two different subjects of the same group (biological replicates). The nearly optimal linear correlation (R2 value ≥ 0.95) and slope (y = 1.08 ± 0.15) close to the theoretical value (1.00) were obtained (Figure S1 of Supplementary Material). The comparison of all lists revelaed that 1478 proteins were shared among the three groups and that 448, 335 and 452 proteins were specific for Wt, He and Ho cohorts, respectively (Figure 1). Moreover, the number of proteins that He and Ho had in common (*n* = 425) was higher than the number of proteins in common between He and Wt (*n* = 115) or Ho (*n* = 205).

Of note, this approach allowed for the identification of many proteins usually difficult to detect, several of which were characterized by anomalous properties in terms of both MW and pI values, as shown in 2D virtual maps (Figure S2) where all the identified proteins were plotted according to their theoretical molecular weight (MW) and isoelectric point (pI), shown in Figure S2. In particular, around 100 proteins had an MW of < 10 or > 200 kDa and a similar number had a pI > pH 10.0. By contrast, very few proteins (about 10) had a pI < pH 4.0.

### 2.2. Clustering and Differential Analysis

**Figure 2:**
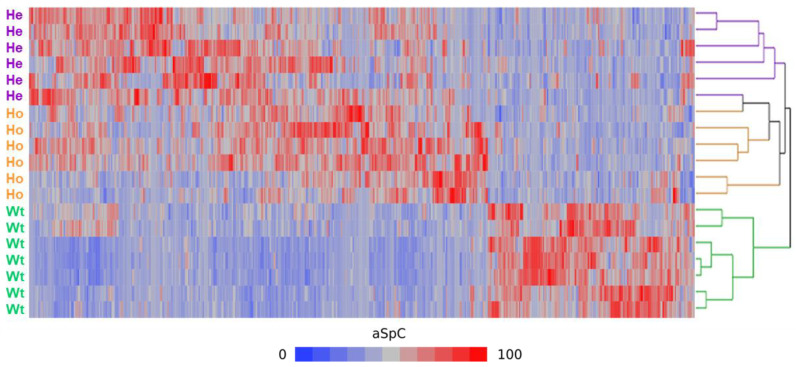
Hierarchical Clustering. Dendrogram of protein lists from 7 Nasu-Hakola subjects and their technical replicates. Clustering was performed by computing the average spectral count (aSpC) value of proteins selected by Linear Discriminant Analysis (LDA); Euclidean’s distance metric and Ward’s methods were applied. Categories were reported in different colours: He subjects in violet, Ho subjects in orange and wt subjects in green.

All 19 lists of proteins identified were compared using the MAProMa platform [14], normalized and used to perform a hierarchical clustering [15]. As shown in Figure 2, the seven subjects were correctly grouped into the three corresponding categories: Wt, He, and Ho. Interestingly, two main branches could be observed: one involved protein lists from Wt only, while the other included both He and Ho. The latter two cohorts were correctly sub-grouped in their set.

The average lists from each category (the number of proteins identified were: 2246, 2353 and 2560 for Wt, He and Ho, respectively) were compared singularly by applying the DAve (Differential Average) and DCI (Differential Coefficient Index) algorithms of the MAProMa Software. Using stringent filters (0.4 and 15, respectively) on aSpC for both Dave and DCI indexes, 192 differentially expressed proteins (DEPs) could be extracted. Among these, 147 distinct proteins were differentially expressed between Wt and Ho: 55 were upregulated in Wt (among which 2, HSP90AB2P and KRT10, were exclusive), whereas 92 were upregulated in Ho. One hundred and sixty proteins were differentially represented in Wt and He: 52 were upregulated in Wt (among which 1, TMSB4X, was exclusive), whereas 108 were upregulated in He and only 28 distinct proteins (13 were upregulated in He and 15 were upregulated in Ho) distinguished the protein profile of He from that of Ho. This minimal difference corroborated with a cluster analysis shown in Figure 2, highlights the homology of protein expression between He and Ho. The complete list of the up- and down-expressed proteins in the different categories is reported in Table S2 of the Supplementary Material.

**Figure 3:**
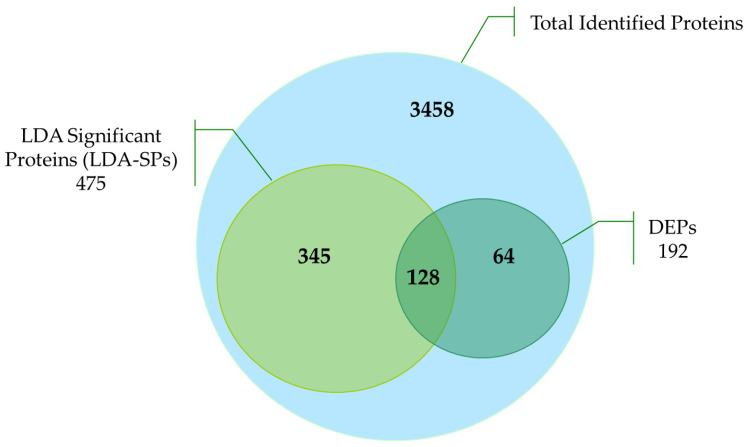
LDA-SPs and DEPs Diagram. The figure shows the portion of LDA Significant Proteins (LDA-SPs) and differentially expressed proteins (DEPs) compared to the total identified distinct proteins in all processed samples and replicates.

The application of the Linear Discriminant Analysis (LDA) allowed the extraction of 475 significant proteins (LDA-SPs, see Table S3) with a *p* < 0.01 from the total of proteins identified [16]. The layout of Figure 3 clearly shows that these selected proteins included 128 of all 192 DEPs mentioned above.

By interrogating UniPROT, it could be observed that 40% of proteins extracted by LDA and label-free differential analysis were cellular; 28% were organelles, 22% were from the macromolecular complex, and 7% were membrane proteins. This subcellular distribution matched with the localization of total identified proteins (Figure S3).

### 2.3. Network Analysis: Systems Biology Evaluation

**Figure 4:**
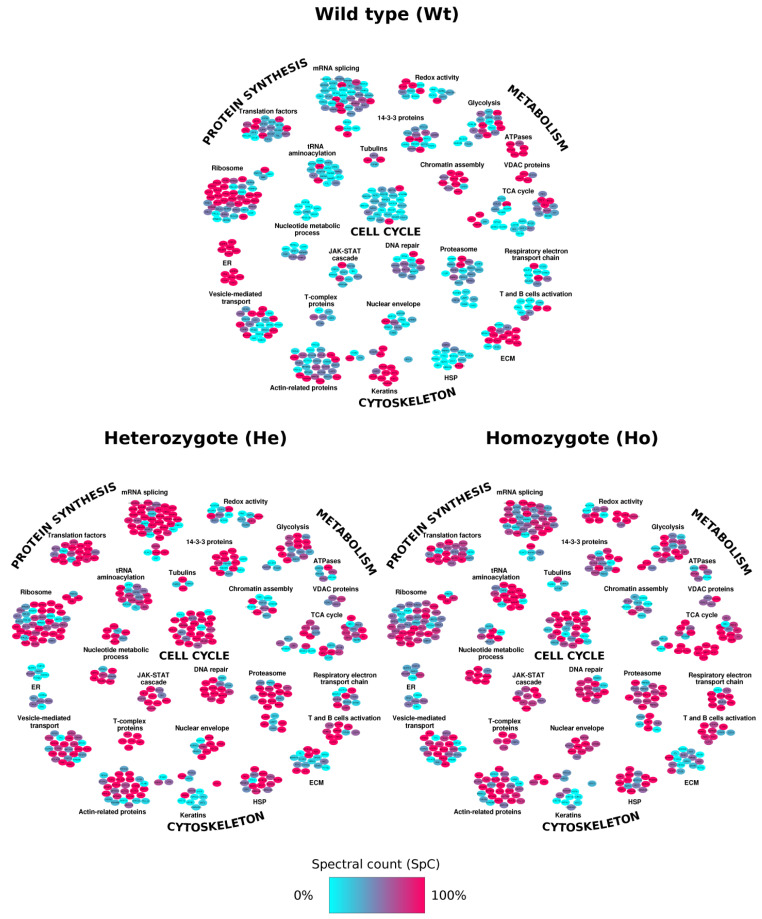
Protein–Protein Interaction (PPI) Network. Interactome network built for Wild type (Wt), Heterozygote (He) and Homozygote (Ho) subjects through the mapping of proteins found to be differentially expressed by LDA and MAProMa analysis. The color code of distinct nodes reflects the normalized SpC values of each examined condition.

The identification of the largest possible number of proteins and the availability of the Homo Sapiens network dataset was the premise of this study; to investigate the functional relationships among proteins anomalous to Wt, He and Ho. Both the differentially expressed proteins and the proteins selected by means of LDA were plotted onto the Protein–Protein Interaction (PPI) Network using Cytoscape and its plug-ins [17,18]. This approach identified the following four principal sub-networks: (i) energetic metabolism (Glycolysis, Krebs Cycle and Electron Transport Chain); (ii) cell cycle (Jak-STAT cascade, Chromatin assembly and Proteasome); (iii) protein synthesis (Translation factors and mRNA splicing) and (iv) cytoskeleton (Actin-related proteins, HSP, T and B cells activation) (Figure 4).

This large-scale identification of proteins allowed for a significant increase in the number of differentially expressed proteins belonging to the glycolysis and Krebs cycle pathways previously detected by Giuliano et al. [11] with the on-gel procedure.

**Figure 5:**
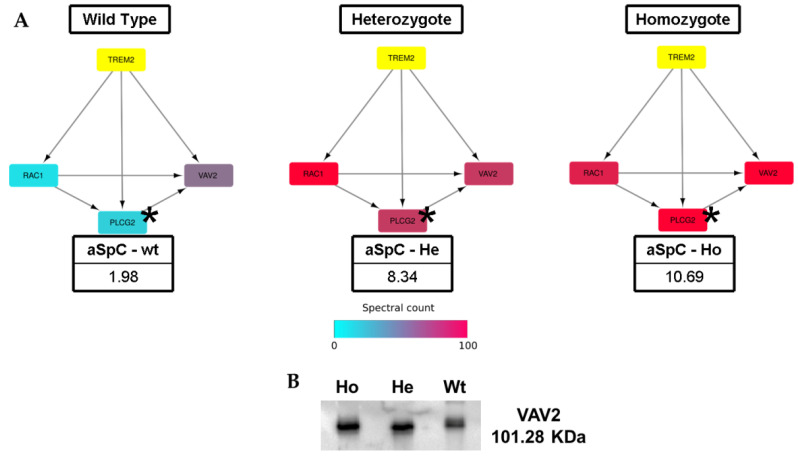
Trem2 Interactor Proteins. (**A**) Three different proteins associated with the TREM2 network were highlighted: RAC1, PLCG2 and VAV2. These interactor proteins were upregulated in NHD patients (Ho) in comparison with healthy subjects (Wt). (**B**) Western blotting of VAV2 protein. * Statistically significant value.

Given the important role played in this pathology by TREM2 receptor, our attention was focused on the identification of this protein. While this protein could not be detected in our proteomic analysis, the identification of three specific proteins that interacted with TREM2-Ras-related C3 botulinum toxin substrate 1 (RAC1), Guanine nucleotide exchange factor (VAV2) and 1-Phosphatidylinositol 4,5-Bisphosphate Phosphodiesterase Gamma-2 (PLCG2), led us to investigate this network in more detail. Of particular interest was the evidence that these TREM2 interactor proteins were differentially expressed in the three groups. The data shown in Figure 5, panel A suggest that their level of expression was gradually increased when moving from Wt to He, the highest upregulation being observed in Ho. The positive correlation between this observation and the Western blot data generated from the comparison of band intensities of VAV2 by using ImageJ software, validated this result, as shown in Figure 5, panel B.

While not succeeding in the detection of DAP12, the co-receptor of TREM2, identification of its protein interactor Sialic acid-binding Ig-like lectin 14 (SIGLEC14) was successful.

## 3. Discussion

This report shows the potential of the MudPIT proteomic platform combined with in-house software and online bioinformatics tools, to identify the proteins expressed in NHD and to characterize the major biochemical pathways involved.

A previous 2-DE proteomic study performed on the lymphoblastoid B cells of two homozygotes in which dementia manifests, four heterozygotes affected by this disorder, and a healthy control of the same family, led to the identification of a few proteins which displayed altered expression among the cohorts [11]. However, due to the inherent limitations of the approach applied, these findings, while being strongly correlated with the data available in the literature, relative to other neurodegenerative disorders [19,20,21,22,23,24], were partial and could not answer all of the questions about the dynamics of NHD. Indeed, the label-free proteomic platform described in this report could help bridge that gap. In fact, the detection of the largest possible number of proteins paves the way for a detailed analysis of the proteome differences among the three conditions examined and for the identification of key protein markers potentially useful for the diagnosis and/or treatment of this TREM2-based disease.

To the best of our knowledge, this is the most complete proteomic analysis of LCLs performed on subjects of an entire family carrier of *TREM2* mutation. Specifically, around 3500 distinct proteins (a number 100-fold higher than that obtained by the previously mentioned 2-DE approach), including proteins with a wide dynamic range in terms of isoelectric point and molecular weight, were identified. It is interesting to note that the data provided by the proteomic clustering allowed us to associate subsets of proteins to each specific cohort (Wt, He or Ho) and to confirm the clinical classification of the subjects investigated, thus offering indirect proof of the efficiency of the MudPIT approach to studies on neurodegenerative diseases (NDs).

To better appreciate the biological message of this work and to visualize the processes involved in the disorder, a PPI Network was built. We observed that, while most of proteins identified were shared among Wt, He and Ho, a few of them were anomalous for each cohort and the profile of Wt differed from those of Ho and He for several differentially expressed proteins (147 and 160, respectively). Interestingly, more than 90% of proteins that showed a specific behavior in Wt subjects appeared to own completely opposite trend in He and Ho. They also exhibited different expression levels; most proteins that were down-represented in sub-networks of Wt were over-expressed in He and Ho subjects. The small number (only 28) of up- or down-regulated distinct proteins that differentiated He and Ho evidenced a strong homology of protein expression between these two cohorts. This finding was of particular interest since, assuming that the He phenotype was healthy, the greater similarity of its proteome profile to Ho than to Wt, while unexpected, provided a novel context for interpreting disease symptoms.

Based on the total human protein network and on the occurring functional relationships, the most significant proteins (DEPs and LDA-SPs) were grouped in four principal sub-networks. Specifically, the high number of differentially expressed proteins involved in energetic metabolism allowed us to validate an assumption previously made: that the impaired glucose metabolism could be the result of the accumulation of glycolytic intermediates [11]. The current experimental data are highly consistent with the hypothesis of an alteration of energy metabolism in NHD patients. This hypothesis supports the idea that the general decrease of energy metabolism due to the reduced metabolic rate of glucose may be a feature of NHD, at least as far as the neurodegenerative aspect is concerned.

Compared with other tissues, the brain is a high energy-demanding organ (it utilizes about 25% of the body’s total glucose) and relies on the efficient production of ATP via the sequence of metabolic pathways (glycolysis, the TCA cycle and oxidative phosphorylation) to support synaptic transmission. It has been reported that glucose metabolism is defective in a variety of neurodegenerative disorders including AD, FTD and Mild Cognitive Impairment [25,26,27]. This defect results from the oxidative damage to key proteins in the aforementioned metabolisms and leads to a decreased ATP intake with the consequent changes in brain function and survival.

Indeed, most glycolytic proteins were upregulated in homozygotes, while proteins related to redox activity, TCA and the Respiratory electron transport chain, were downregulated in heterozygote. Among the proteins identified, both ALDOC and ENO1 have been previously associated with psychiatric disorders [28,29,30]. ALDOC is a brain-specific glycolytic enzyme that, in agreement with previous reports on mood disorders [31,32], was found to be upregulated in diseased subjects.

The upregulation of the proteins implicated in the cell cycle, JAK-STAT cascade [33], DNA repair and proteasome in both He and Ho, confirmed the effects of *TREM2* mutations on cell proliferation [34,35]. These findings were coherent with the observed downregulation of the chromatin assembly sub-network and the activation of actin-related proteins and HSP processes. In particular, the sub-network of heat shock proteins (HSP) were completely down-expressed in healthy subjects in comparison with He and/or Ho. This observation agrees with the results reported by Stefano at al. [36] about the importance of HSP60 (also called HSPD1) as an activation agonist of TREM2 through the induction of all critical processes (phagocytosis, proliferation, activation and migration, and apoptosis) governed by the receptor in myeloid cells, osteoclasts, and microglia. The upregulation of HSP60 was also reported by Koh et al. [37] in a proteomic work investigating the implication of this protein in osteoclast bone resorption. Our data are also supported by a recent work by Ferrari et al. [38], who reported in FTD individuals a co-expression network of FTD cluster genes that revealed a specific enrichment for DNA metabolism (i.e., transcription regulation and chromatin remodeling), immune processes and protein meta/catabolism.

Of note, the recognition of the role of an innate immune activation in the pathogenesis of many neurodegenerative diseases, including Parkinson’s disease (PD), amyotrophic lateral sclerosis (ALS), Huntington’s disease (HD) and frontotemporal dementia (FTD), is growing [39]. It is interesting to note that the *TREM2* gene is allocated on chromosome 6, where 100 genes codifying the immunological system are organized. In this context, the sub-network related to T- and B-cell activation were anomalously upregulated in He subjects. In particular, NCKAP1L, CD74 and LCP1 were upregulated in He vs. Ho while only CD38 was upregulated in diseased subjects. An increase in the CD74 protein, a chaperone involved in antigen presentation during immune response, was associated with an increase in the neurodegeneration process. Furthermore, in studies on patients with diabetes, CD74 was also identified as a microglial activation marker [40,41]. Given the role of CD38 in neuroinflammation and repair processes, Blacher et al. [42] investigated the effect of its deletion on AD pathology and, in agreement with our data, they demonstrated that CD38 mice exhibited significant reductions in Aβ plaque in AD.

Finally, to evaluate the behavior of TREM2’s activation method in diseased vs. healthy conditions, the investigation was focused on TREM2’s protein network and on some dysregulated proteins involved in TREM2 activation among those identified in the groups investigated (Wt, He, and Ho). Three proteins associated with the TREM2 network were identified, although at low levels: Ras-related C3 botulinum toxin substrate 1 (RAC1), Guanine nucleotide exchange factor (VAV2), and 1-Phosphatidylinositol 4,5-Bisphosphate Phosphodiesterase Gamma-2 (PLCG2). In particular, PLCG2 was a crucial enzyme in transmembrane signalling and in osteoclasts, since it formed a complex with the regulatory adapter molecule GAB2, modulating GAB2 recruitment to RANK and inducing osteoclastogenesis [43]. It is interesting to note that the three identified proteins showed a positive trend, increasing their expression at similar levels in both He and Ho. The fact that NHD occurs in Ho, may only be considered a confirmation that PLCG2, RAC1 and VAV2 were not correlated to the disease. While DAP12, the co-receptor of TREM2, was not detected in our analysis, it was possible to identify one of its interactors, Sialic acid-binding Ig-like lectin 14 (SIGLEC14), an additional protein linked to the chaperonin pathway.

The data reported at present support the hypothesis that events occurring in LCLs could mirror the events in the districts where the pathology is localized, e.g., microglia and osteoclast cells.

### Limitations and Advantages of This Study

A limitation of the present study could be the sample size of the individuals examined. However, the apparently low number of subjects enrolled in this study (*n* = 7; 2/7 kin NHD subjects), considering the rare pathology that they are suffering, is a strength when compared with the individual cases so far discussed in the literature [44].

It may be also argued that the use of the Lymphoblastoid cells line (LCL) to investigate a pathology that manifests its symptoms in districts (brain and bones) different from blood, represents a limitation of the study. However, it is important to note that, together with human-induced pluripotent stem cell (iPSC)-derived neurons, human LCLs have been employed in biomedical research for decades, and they are increasingly being used as in vitro research tools for personalized medicine for the treating of brain diseases [45,46].

In our case, their use brought more advantages than limitations, especially considering the rarity of the disease and the inability to proceed with frequent sampling on NHD patients.

The preparation of human LCLs is simple, inexpensive and reliable, and could derive from a great number of peripheral blood lymphocytes, resulting in the polyclonal nature of cell lines. In fact, they can be maintained in continuous in vitro growth over many months and their genome remains stable during subsequent cell divisions. By contrast, is the phenotype of these cells that is likely to be considered a pitfall of the use of B-lymphocytes. However, based on the proof reported by Giuliano et al. [11] that LCLs express the homozygous mutation of the *TREM2* gene, the decision was made to select and immortalize a sub-group of cells derived from blood to be investigated in the present work.

## 4. Materials and Methods

### 4.1. Subjects

All individuals considered in this study belonged to the same family native to a restricted area of northern Italy. The family consisted of seven members: two patients with homozygous C-to-T mutation at position 97 in exon 2 of *TREM2* gene (Ho); four healthy carriers with heterozygous mutation (He); and one healthy individual (Wt). The family pedigree is reported by Giuliano et al. [11]. Based on the available data, it was possible to exclude the consanguinity in the last five generations. Moreover, during the early 2000s, all subjects were submitted to neuropsychological tests which covered a wide range of cognitive functions. The complete medical report was described by Soragna et al. [47]. The Ethics Committee of the Neurological Institute “C. Mondino”, Pavia (Italy) and the “Laura Fossati Foundation”, Montesegale, Pavia (Italy), reviewed and authorized studies on these patients.

### 4.2. Lymphoblastoid B-Cell Line and Protein Extraction

The proteomic analysis described in this study was performed on Lymphoblastoid cell lines (LCLs) immortalized from B-Lymphocytes collected from each subject, as previously detailed by Giuliano et al. [11]. For each subject, about 30 × 106 Lymphoblastic B-cells were used. Cells were maintained in suspension culture in RPMI 1640 medium supplemented with 10% fetal bovine serum, 4 mM glutamine, streptomycin, and penicillin. To obtain total extracts, cells were harvested by centrifugation (13,006× *g* for 5 min at 4 °C), resuspended into 100 μL of 0.1 M NH_4_HCO_3_ pH 8.0, and homogenized. The pellets were treated with 0.25% (*v/v*) of MS-compatible detergent (Rapigest^TM^ SF, Waters Co., Milford, MA, USA) and incubated for 20 min at 100 °C [48]. Finally, mixtures were chilled to room temperature and after centrifugation (2.2 g for 10 min at room temperature) the supernatant was recovered. Protein concentration was determined by using the SPN^TM^-Protein assay (G-Biosciences, St. Louis, MO, USA) kit. Each sample was then digested overnight at 37 °C by adding sequencing grade-modified trypsin (Promega, Madison, WI, USA) at an enzyme/substrate ratio of 1:50 (*w/w*). An additional aliquot of trypsin at 1:100 ratio (*w/w*) was added in the morning and digestion was then prolonged for 4 h. The addition of 0.5% trifluoroacetic acid stopped the enzymatic reaction and subsequent incubation at 37 °C for 45 min completed the acidic hydrolysis of RapiGest. The water-insoluble degradation products were removed by centrifuging at 13,000× *g* for 10 min and supernatants containing the resulting peptide mixtures were desalted with Pierce^TM^ C-18 spin columns (Thermo Fisher Scientific, San Josè, CA, USA) and resuspended in 0.1% (*v/v*) formic acid (FA).

### 4.3. LC-MS/MS Analysis

The whole proteome of samples under investigation was produced by applying the multidimensional protein identification technology (MudPIT) [12,13]. This consisted of a 2dLC-MS/MS platform, composed of a two dimensional micro-high performance liquid chromatography system (Surveyor HPLC; Thermo Fisher Scientific, Inc., San Jose, CA, USA) coupled online to a mass spectrometer, using ProteomeX-2 configuration (Thermo Fisher Scientific, Inc.).

Briefly, the peptide mixture (5 μg) was loaded onto a strong cation exchange column (PolyLC-SCX 0.3 i.d. × 100 mm, 5 µm, 300 Å, PolyLCINC, Columbia, MD, USA); eluted stepwise with ammonium chloride injections of increasing molarity (10, 20, 40, 80, 120, 200, 400, 600, 700 mM) and captured in turn onto peptide traps (Zorbax 300 SB-C18, 0.3 i.d. × 5 mm, 5 µm, 300 Å; Agilent Technologies, Santa Clara, CA, USA) for concentration and desalting prior to further separation on a reverse phase C18 column (Biobasic-C18, 0.18 i.d. × 100 mm, 5µm, 300 Å, Thermo Fisher Scientific, CA, USA). Peptides were gradually eluted from this column using a 65 min linear gradient from 5 to 95% acetonitrile (ACN) containing 0.1% FA. Flow rate was set at 130 μL/min and was split to achieve a final flux of 2 μL/min at the end of the column.

Then, eluting peptides were electrosprayed directly into an LTQ-OrbitrapXL mass spectrometer (Thermo Fisher Scientific, Massachusetts, CA, USA) equipped with a nanospray ion source. The spray capillary voltage was set at 1.7 kV and the ion transfer capillary temperature was held at 220 °C. For each step of peptide elution from the C18 column, full MS spectra were recorded over a 400–1600 *m/z* range in positive ion mode, with a resolving power of 60,000 FWHM (full width at half-maximum) and a scan rate of 2 spectra. This step was followed by five low-resolution MS/MS events that were sequentially generated in a data-dependent manner for the top five ions selected from the full MS spectrum, using dynamic exclusion of 0.5 min for MS/MS analysis when a peptide ion was analysed twice. In particular, the MS/MS scans were acquired by CID fragmentation, setting a normalized collision energy of 35% on the precursor ion. Mass spectrometer scan functions and high-performance liquid chromatography solvent gradients were controlled by the Xcalibur data system version 1.4 (Thermo Fisher Scientific, Massachusetts, CA, USA).

As described in the previous paragraph, three different groups of samples were examined in this study: 2 Ho subjects, 4 He subjects and only 1 WT subject. Therefore, to obtain a significant and, at the same time, a comparable number of data for each category, more technical and/or biological replicates were considered. In fact, each of the two Ho samples was injected three times, two of the four He samples were injected twice, while the only available WT sample was injected seven times, producing a total of 19 protein lists. The MS data were deposited to the ProteomeXchange Consortium via the PRIDE [49] partner repository (ftp://massive.ucsd.edu/MSV000087622/ accessed on 3 July 2021).

### 4.4. Data Handling and Protein Profile of LCLs

Data handling was performed using the 3.3.1 SP1 Bioworks version based on SEQUEST algorithm (University of Washington, licensed to Thermo Fisher San Jose, CA, USA) [50]. The experimental MS/MS spectra were correlated to tryptic peptide sequences by comparison with the theoretical mass spectra obtained by in-silico digestion of the Homo sapiens protein database (about 228763 entries), downloaded January 2013 from the National Centre for Biotechnology Information (NCBI) website (http://www.ncbi.nlm.nih.gov accessed on 3 July 2021).

The following criteria were used for the identification of peptide sequences and related proteins: trypsin as enzyme, three missed cleavages per peptide were allowed, and mass tolerances of 50 ppm for precursor ions and 0.8 Da for fragment ions were used. Moreover, to assign a final score to proteins, the SEQUEST output data were filtered by setting the peptide probability to 1 × 10^−3^, the minimum correlation score values (Xcorr) were chosen as greater than 1.5, 2.0, 2.5, and 3.0 for single-, double-, triple-, and quadruple-charged ions respectively, and a consensus score higher than 10. Validation based on separate target and decoy searches and subsequent calculation of classical score-based false discovery rates (FDR) were used for assessing the statistical significance of the identifications. False-positive peptides ratio, calculated through reverse database, was less than 1%. For decoy searches a reversed version of the target human protein database was generated using the reverse database function in Bioworks 3.3.1 software (Thermo Fisher Scientific, Waltham, MA, USA).

To convert the NCBI Accession code of the identified proteins into Gene Name, an in-house script was realized in Python programming language. In addition, the GI accession numbers of identified proteins were correlated to those downloaded January 2019 from the UniProt repository (http://www.uniprot.org/ accessed on 3 July 2021).

Individual cellular location was assigned to each protein according to the UniProt database. It should be noted that some proteins may have multiple cellular locations: in these cases, the most typical and representative cellular location was manually assigned. Proteins that could not be assigned a cellular location were not included.

### 4.5. Label-Free Differential Analysis

To improve the identification of differentially expressed proteins, two different and complementary label-free approaches were adopted: an in-house algorithm, Multidimensional Algorithm Protein Map (MAProMa) [14,15] and Linear Discriminant Analysis (LDA) [16].

#### 4.5.1. MAProMa

The 19 protein lists obtained from the SEQUEST algorithm were aligned and compared by means of the average spectral counts (aSpC) [15], corresponding to the average of all the spectra identified for a protein and, consequently, to its relative abundance, in each analyzed condition (Ho, He, and Wt). In depth, to select differentially expressed proteins, the three subgroups were pairwise compared (He vs. Ho; He vs. Wt; Wt vs. Ho), applying a threshold of 0.4 and 15 on the two MAProMa indexes DAve (Differential Average) and DCI (Differential Confidence Index), respectively. DAve, which evaluates changes in protein expression, was defined as (X − Y)/(X + Y)/0.5, while DCI, which evaluates the confidence of differential expression, was defined as (X + Y) × (X − Y)/2. The X and Y terms represented the SpC of a given protein in two compared samples.

#### 4.5.2. Linear Discriminant Analysis and Hierarchical Clustering

To reduce data dimensions, protein lists obtained by MudPIT replicate analyses were processed by means of Linear Discriminant Analysis (LDA) [16]. Specifically, a matrix mxn consisting of 3458 proteins and 19 replicates grouped into three subgroups (Ho, He, Wt), was considered. LDA was applied by using a common covariance matrix for all groups and the Mahalanobis distance [51] from each point to each group’s multivariate mean (proteins derived from the same gene were grouped). To select proteins that distinguished the subgroups analyzed, those with the largest F ratio (>3.4) and the smallest *p*-value (<0.05) were considered. Specifically, the F ratio represented the model mean square divided by the error mean square, whereas the *p*-value indicated the probability of obtaining an F value greater than that calculated, provided that no difference could be observed between the population group means. The RapidMiner software was used.

All 19 lists were evaluated by means of unsupervised learning methods, such as hierarchical clustering [52], using in-house R-scripts, based on the XlsReadWrite, and clue and clValid libraries (http://cran.r-project.org downloaded 3 January 2013). In particular, the Euclidean distance metric was applied, and an agglomerative coefficient was calculated (Agglomerative Nesting—AGNES).

### 4.6. Network Analysis

A Global Mammalian Protein Interactomic (GMPI) network was built by means of Cytoscape [17,18,53] and its plugin, combined data retrieved from major interactomic repositories, including HPRD, MINT, BioGrid, IntAct, DIP, BIND and Pathway Commons online database. In addition, it was complemented by in-house manually curated data derived from literature. The combined dataset included only manually curated protein–protein binary interactions inferred by two to six independent methods. Functional, protein–DNA, protein–RNA, protein–metabolite and protein–drug interactions, as well as duplicates and self-interactions that could lead to miscalculations of topological parameters, were removed.

Briefly, the human PPI network retrieved by PESCA plugin was matched to all differentially expressed proteins and a subnetwork of 469 nodes and 5628 interactions was obtained. This network was evaluated at topological and functional level to identify topological and functional modules/clusters. In particular, MCODE [54] plugin was used to find highly interconnected regions in the network, while BINGO 2.44 [55] plugin was used to evaluate the most represented GO terms: Homo sapiens organism, hypergeometric test, Benjamini & Hochberg FDR correction, and a significance level ≤0.001 were applied.

### 4.7. Western Blotting

To validate TREM2-interactor protein VAV 2, Western blotting analysis was carried out. A weight of 50 µg of proteins from Wt, He and Ho were separated on mini-PROTEAN TGX stain-free Precast gels for SDS-PAGE (8–16% gradient). At the end of the run, proteins were transferred onto PVDF membranes by means of a Trans-Blot Turbo Transfer System (Bio-Rad), applying up to 2.5 V for 7 min. After 1h incubation in 5% BSA/milk (10 mL) in TBS/PBS and three washes with TBST/PBST (0.1% Tween in 10 mL), the membranes were incubated overnight in 1% BSA/milk (at 4 °C) with the relative monoclonal antibodies (Thermo Fischer/Abcam, Waltham, MA, USA) at a dilution of 1:5000. After washing the membranes three times with TBST/PBST (10 mL), incubation with the second antibody (polyclonal anti-rabbit/anti-mouse immunoglobulin) diluted 1:8000 (Cell Signaling Technology, Danvers, MA, USA) was performed in 1% TBST/PBST for 1 h at room temperature for each Western blot. The membranes were finally washed three times with TBST/PBST and incubated in ECL Prime Solution (GE, Healthcare, Chicago, IL, USA). Immunoblots were acquired with the ImageQuant LAS 4000 analyzer (GE Healthcare, Chicago, IL, USA).

### 4.8. Statistical Analysis

Identified proteins were evaluated by LDA (F ratio > 3.4 and a *p*-value < 0.05) [15] and MAProMa (confidence thresholds were set as DAve ≥ |0.4| and a DCI ≥ |15|) [16] platforms.

## 5. Conclusions

Nasu-Hakola Disease, while being a very rare pathology, can be considered, due to its anomalous features, as a good model for a deeper understanding of Frontotemporal TREM2-based diseases. Given the absence of reports dealing with the application of shotgun proteomics to NHD investigation, this work represents a proof of principle in this field. The combination of 2D-LC and MS/MS technologies, in a completely automated way, allows for an extensive characterization of complex biological matrices and does not suffer from interferences exerted by highly expressed proteins (even in a wide dynamic range of protein expression), that may affect the detection of biomarkers, usually present in low amounts.

This approach was useful to characterize metabolic pathways potentially involved in functional alterations of this pathology, as a model for studying the effect of neurological disorders. Sure enough, for the first time, a complete set of expressed proteins by LCLs belonging to similar NHD subjects was investigated.

Moreover, our results open the way to new investigations aiming to unravel the reasons for the differences between healthy carriers and sick subjects. By means of network analysis, the possible metabolic pathways involved in functional alterations caused by NHD were widely characterized. In addition, our study may help to set up new experiments to identify possible circulating biomarkers. This work contributed to obtaining a broader understanding of the protein perturbations involved in NHD, a starting point for the discovery of new biomarkers of TREM2-based dementias. Given the similarity with other FTDs, our approach could significantly contribute, in the future, to managing these diseases. In fact, this approach could represent a new non-invasive early diagnostic method to investigate neurodegenerative diseases, for which only post mortem analyses are feasible.

Of course, more investigations are needed to confirm the correlation of the described pathways with the TREM2-related loss of functions disease, including frontotemporal dementia, but our approach has aptly demonstrated that it can contribute to managing NHD and other FTD diseases in the future. Our main focus was the discovery of potential biomarkers which have the ability to diagnose these diseases early on or to characterize different pathological phenotypes, to unravel new underlying molecular pathways, and to monitor patient responses to new therapies.

## Data Availability

Proteomic data are stored in the ProteomeXchange Consortium repository and are available on request at the following url: ftp://massive.ucsd.edu/MSV000087622/ accessed on 3 July 2021.

## Supplementary Materials

The following are available online at https://www.mdpi.com/article/10.3390/ijms22189959/s1.
