# Supplementary material for: A Shotgun Proteomic Platform for a Global Mapping of Lymphoblastoid Cells to Gain Insight into Nasu-Hakola Disease
Source: Int J Mol Sci. 2021 Sep 15;22(18):9959. doi: 10.3390/ijms22189959 (PMC8472724; doi:10.3390/ijms22189959)
Supplement: Supplementary file 1 [file ijms-22-09959-s001.zip › De Palma A et al_ Supplementary Materials.pdf]

International Journal of
Molecular Sciences

MDPI

De Palma A et al. Supplementary Figures

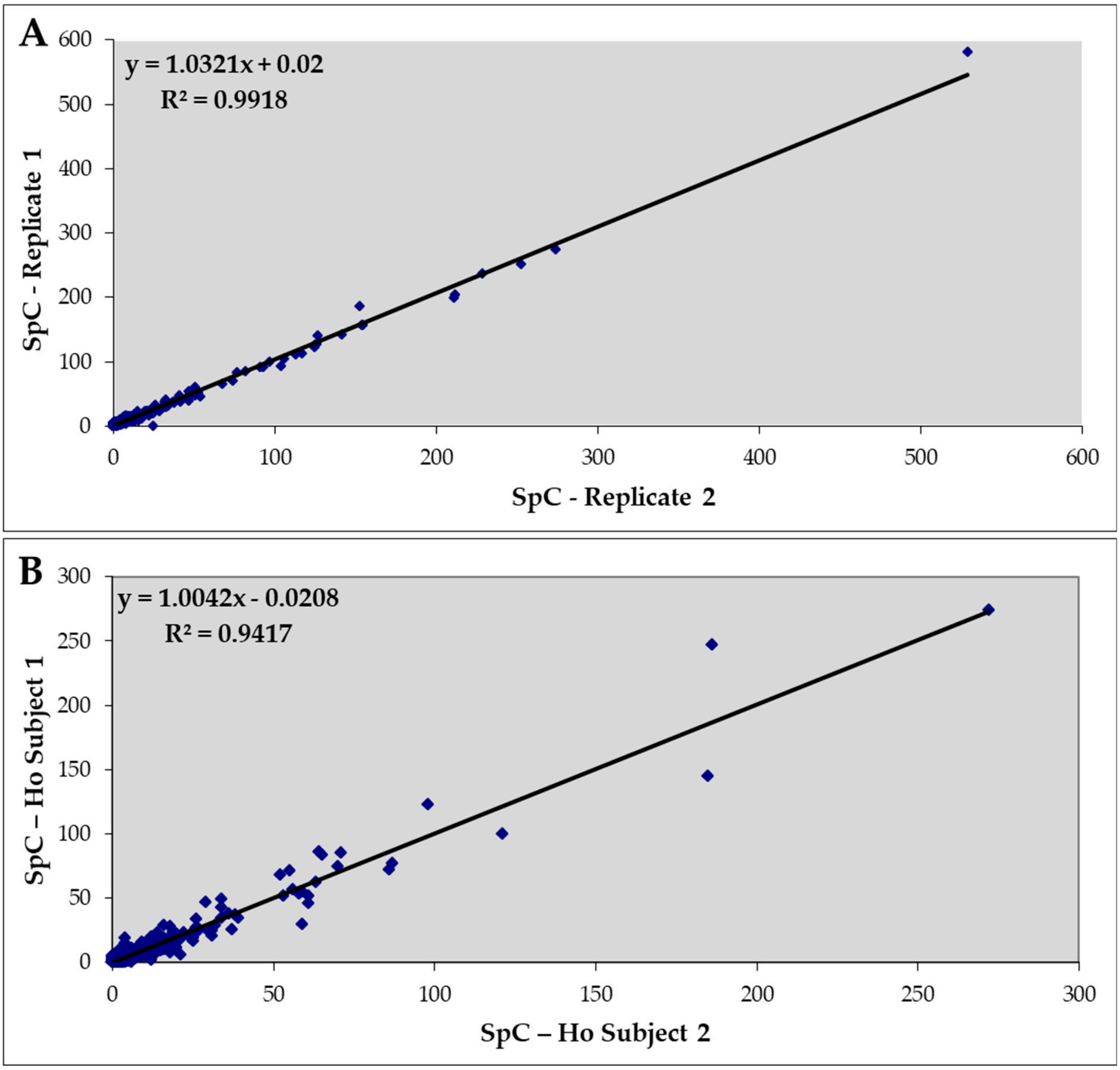

Figure S1. MudPIT Technical and Biological Repeatability. Linear regression analysis obtained by considering SpC values of proteins identified into two technical replicates **(A)** and two Homozygote subjects **(B)** of MudPIT analysis with R^2 and slope (y) close to theoretical value of 1.

Fig S2

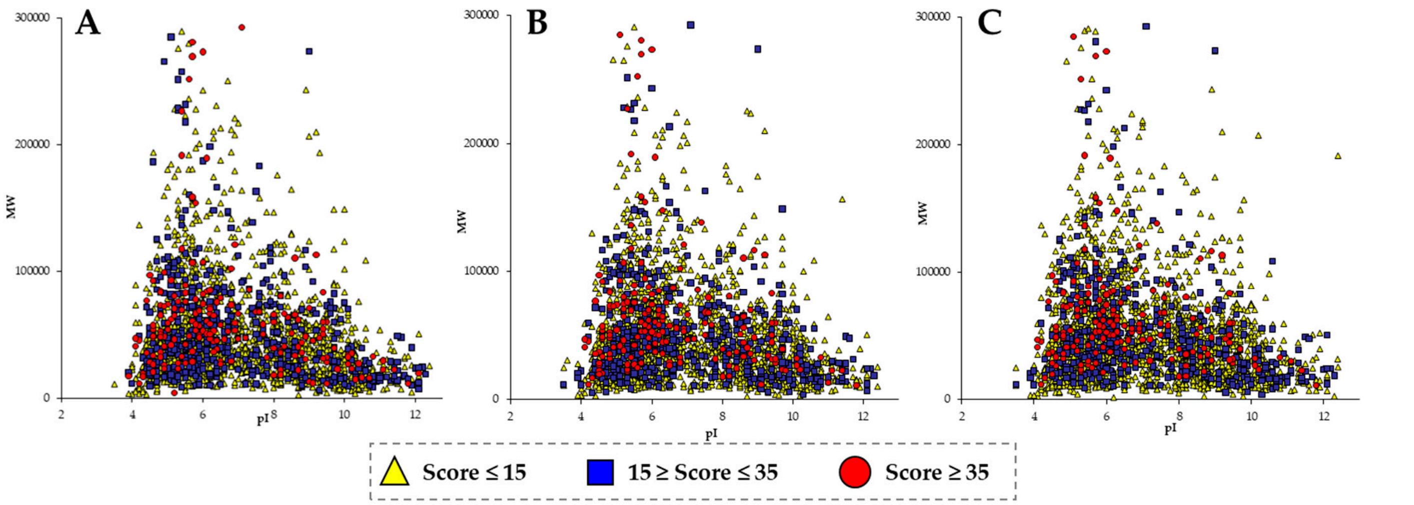

Figure S2. 2D virtual map of Wt (A), He (B) and Ho (C) plotted by MAProMa software. Identified proteins were plotted according to their theoretical pI and MW and for each protein, a color/shape code was used according to the frequency of identification among the samples (proteins with score ≤15 were reported as yellow triangle; proteins with score ≥35 were reported as red circle and proteins that with a score in the range 15-35 were plotted as blue squares.

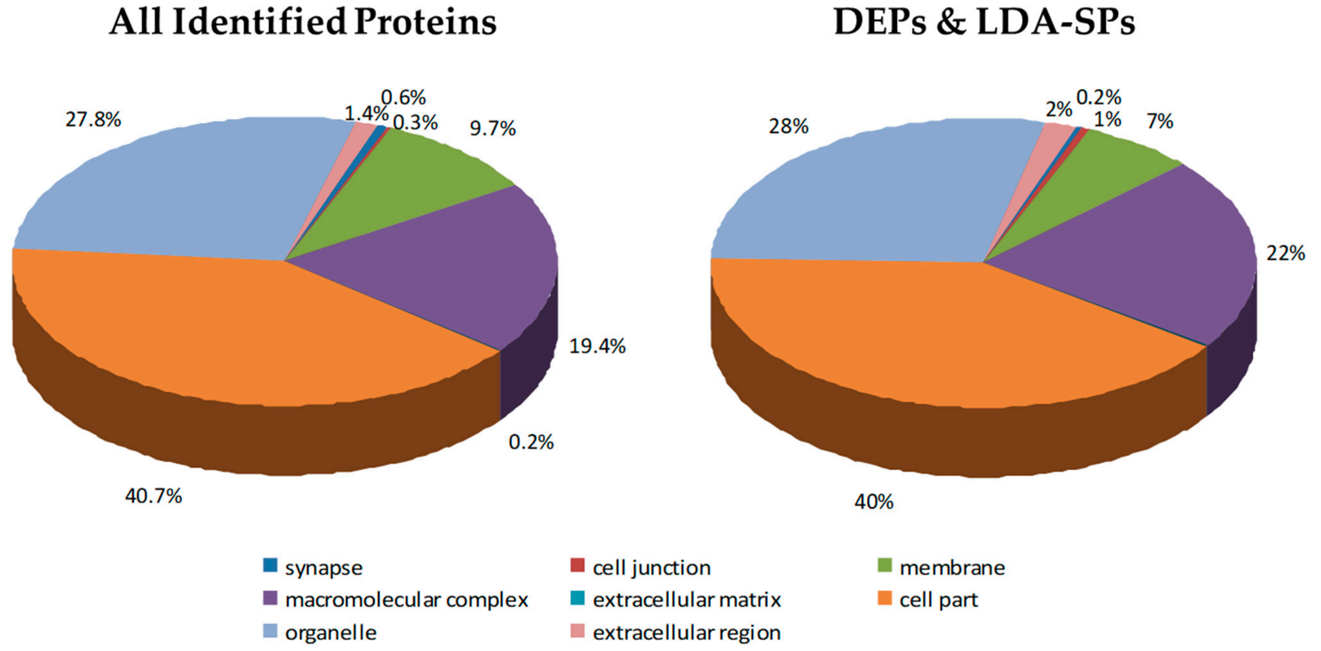

Figure S3. Sub-cellular Localization using data deposited in UniPROT. The protein distribution of DEPs and LDA-SPs matched with the total identified proteins allocation.

De Palma A et al. Supplementary Tables

Table S1. Complete list of the distinct proteins detected in total extracts of Lymphoblastic B-cells stratified in the three distinct conditions under investigation: healthy individual (Wt), healthy carriers with heterozygous mutation (He) and patients with homozygous C-to-T mutation at position 97 in exon 2 of TREM2 gene (Ho). For each protein are reported: NCBI Accession (GI number), Uniprot Accession, Gene name, Reference, pI and MW, Frequency, SpC and Score. Frequency indicates how many times a given protein has been identified under each condition examined. The asterisk next to SpC and Score indicates that the average values are given for each protein of the same condition. The last two columns highlight proteins that were found differentially and/or discriminant expressed, applying the filtering criteria shown in Materials & Methods section. See the supplementary table file attached (De Palma A et al_Table S1).

Table S2. Complete list of differentially expressed proteins between the three conditions examined. For each protein are reported: NCBI Accession (GI number), Uniprot Accession, Gene name, Reference, pI, MW, Frequency (Freq), SpC, DAve and DCI. Frequency indicates how many times a given protein has been identified under each condition examined. The asterisk next to SpC indicates that the average values are given for each protein of the same condition. Three comparison are considered; Wt vs Ho, Wt vs He and He vs Ho. Positive values for DAve and DCI indicate that the protein is more abundant in the first condition, negative values in the second ones. For further details regarding the meaning and the confidence range applied to DAve and DCI see Materials & Methods section. See the supplementary table file attached (De Palma A et al_Table S2).

Table S3. Complete list of the descriptors identified by LDA. For each protein are reported: NCBI Accession (GI number), Uniprot Accession, Gene name, Reference, pI, MW and average SpC values in the three examined conditions (Wt, He and Ho), from which were also calculated F ratio and p-value. For further details regarding the meaning and the confidence range applied to see Materials & Methods section.Complete list of differentially expressed proteins in fetal hAFS-EVs versus perinatal hAFS-EVs. See the supplementary table file attached (De Palma A et al_Table S3).
