# Supplementary figures and images for: A Shotgun Proteomic Platform for a Global Mapping of Lymphoblastoid Cells to Gain Insight into Nasu-Hakola Disease
Source: Int J Mol Sci. 2021 Sep 15;22(18):9959. doi: 10.3390/ijms22189959 (PMC8472724; doi:10.3390/ijms22189959)

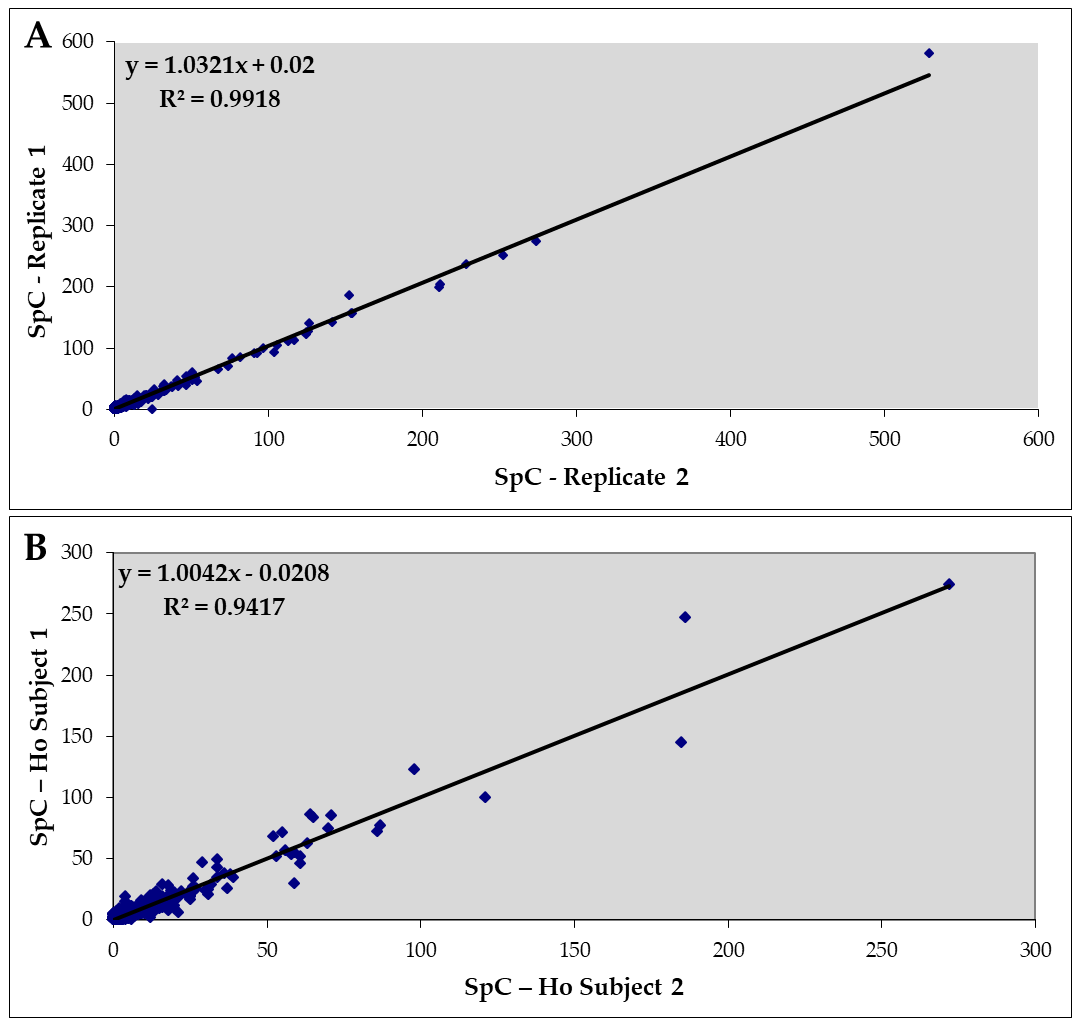

Supplement: Supplementary file 1 [file ijms-22-09959-s001.zip › De Palma A et al_Figure S1.png]

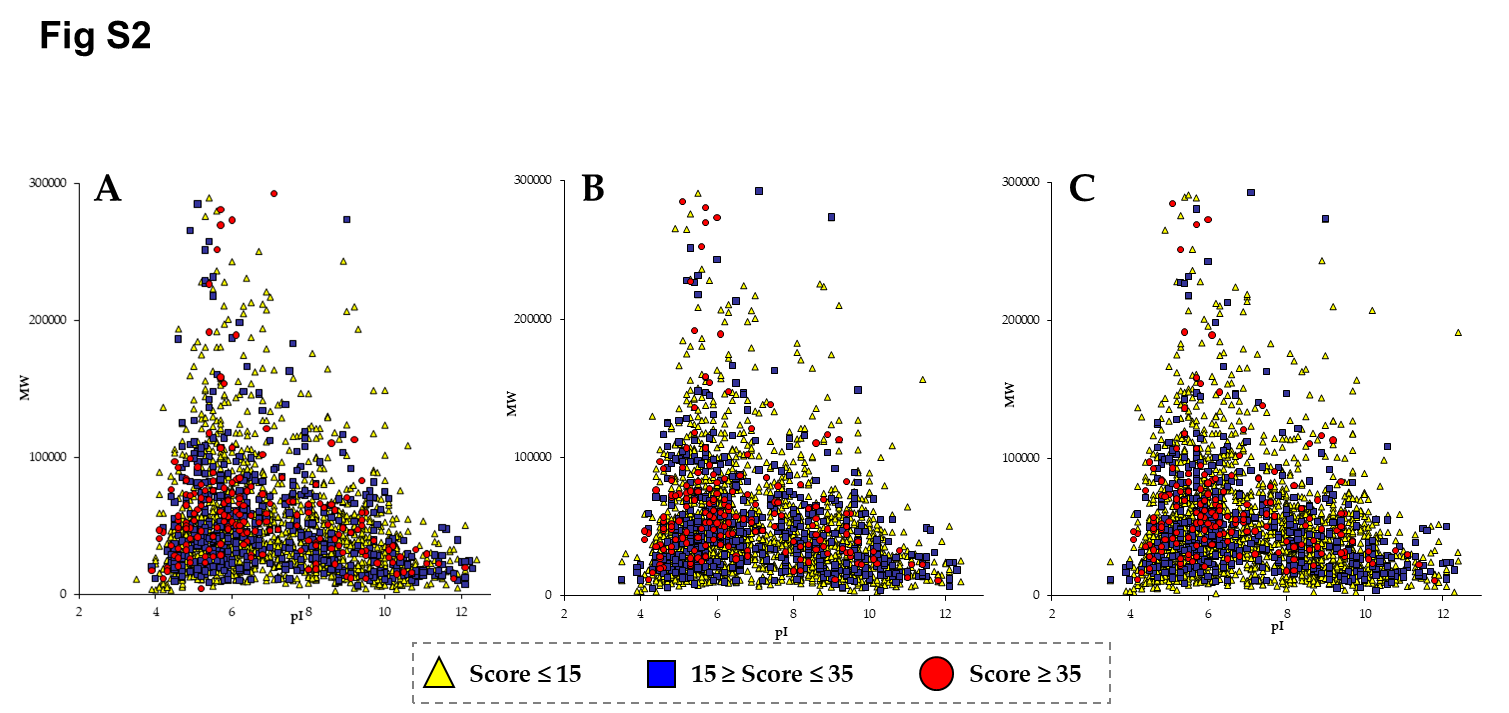

Supplement: Supplementary file 1 [file ijms-22-09959-s001.zip › De Palma A et al_Figure S2.png]

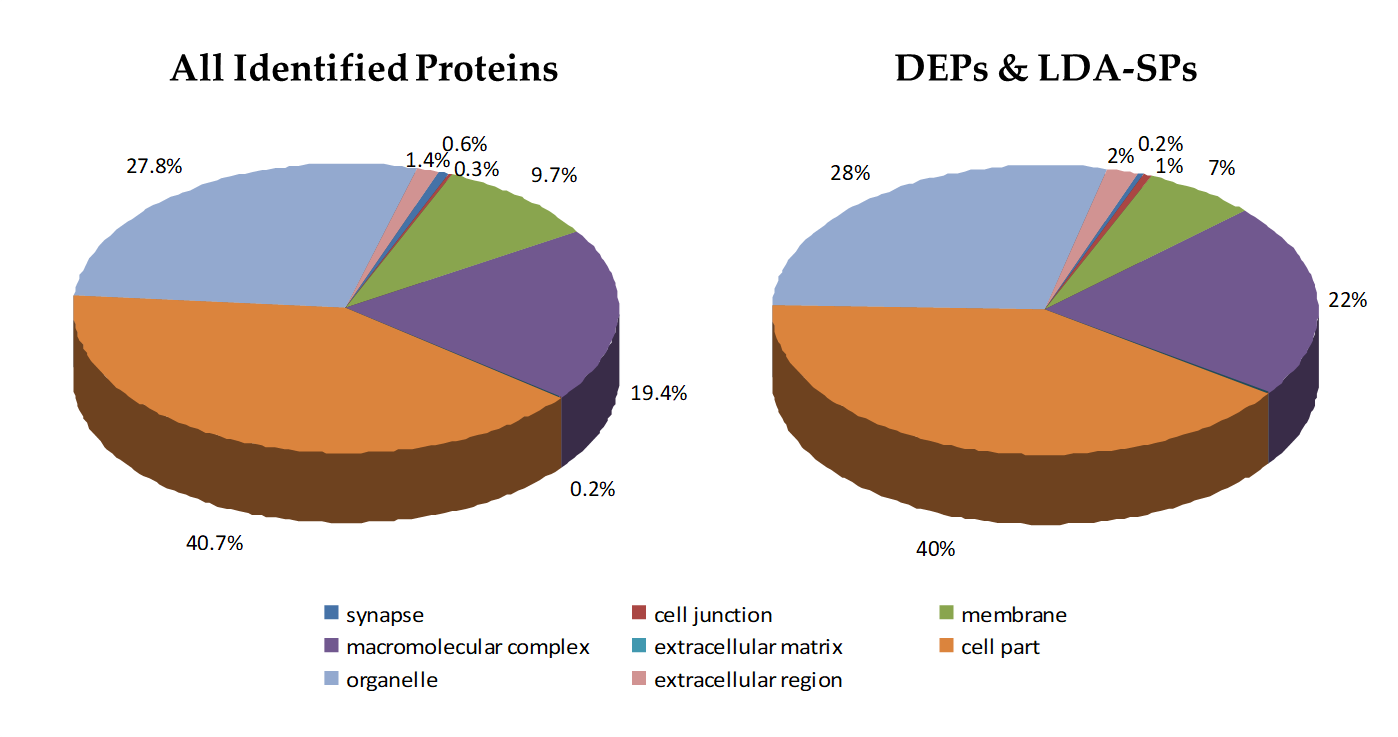

Supplement: Supplementary file 1 [file ijms-22-09959-s001.zip › De Palma A et al_Figure S3.png]
